# Investigation of Structural Dynamics of Enzymes and Protonation States of Substrates Using Computational Tools

**DOI:** 10.3390/catal6060082

**Published:** 2016-05-31

**Authors:** Chia-En A. Chang, Yu-Ming M. Huang, Leonard J. Mueller, Wanli You

**Affiliations:** Department of Chemistry, University of California, Riverside, CA 92521, USA

**Keywords:** force field, calculation, energy, substrate binding

## Abstract

This review discusses the use of molecular modeling tools, together with existing experimental findings, to provide a complete atomic-level description of enzyme dynamics and function. We focus on functionally relevant conformational dynamics of enzymes and the protonation states of substrates. The conformational fluctuations of enzymes usually play a crucial role in substrate recognition and catalysis. Protein dynamics can be altered by a tiny change in a molecular system such as different protonation states of various intermediates or by a significant perturbation such as a ligand association. Here we review recent advances in applying atomistic molecular dynamics (MD) simulations to investigate allosteric and network regulation of tryptophan synthase (TRPS) and protonation states of its intermediates and catalysis. In addition, we review studies using quantum mechanics/molecular mechanics (QM/MM) methods to investigate the protonation states of catalytic residues of β-Ketoacyl ACP synthase I (KasA). We also discuss modeling of large-scale protein motions for HIV-1 protease with coarse-grained Brownian dynamics (BD) simulations.

## 1. Introduction

Conformational changes of enzymes are often related to regulating and creating an optimal environment for efficient chemical catalysis. The dynamics and conformational changes in enzymes may range from the fluctuation of side chains to large-scale protein motions. The former helps adjusting the environment for chemical reactions and the latter enables the protein to create a tertiary structure for substrate binding. The internal motions of proteins may serve as a “gate” in some systems, such as HIV-1 protease, which controls ligand–protein association [1]. In many enzymes or enzyme complexes, protein motions involve allosteric communication to coordinate the function and reactions, which may be intrinsic to many enzymes [2,3]. Residues in the active site directly involved in chemical catalysis may even change allosteric networks in different states during catalysis; example states are a ligand-free resting state and a substrate-bound working state.

In addition to conformational rearrangements of protein side-chains and substrates, protonation states of substrates and key catalytically important residues play critical roles in chemical reactions. For β-Ketoacyl ACP synthase I (KasA), the enzyme that involved in fatty acid synthesis, changes in protonation states on key catalytic residues at different steps are essential in enzyme activity [4,5]. The protonation states may significantly affect protein motions as well. With the cofactor pyridoxal-5′-phosphate (PLP), the bioactive form of vitamin B6, the protonation states of different sites on the coenzyme, such as the phenolic oxygen and PLP ring nitrogen in particular, are thought to be critical for establishing the efficient reaction pathway [6–9]. Moreover, changing a single proton location may interrupt the overall protein–substrate stability and restrain enzyme catalysis.

**Figure 1:**
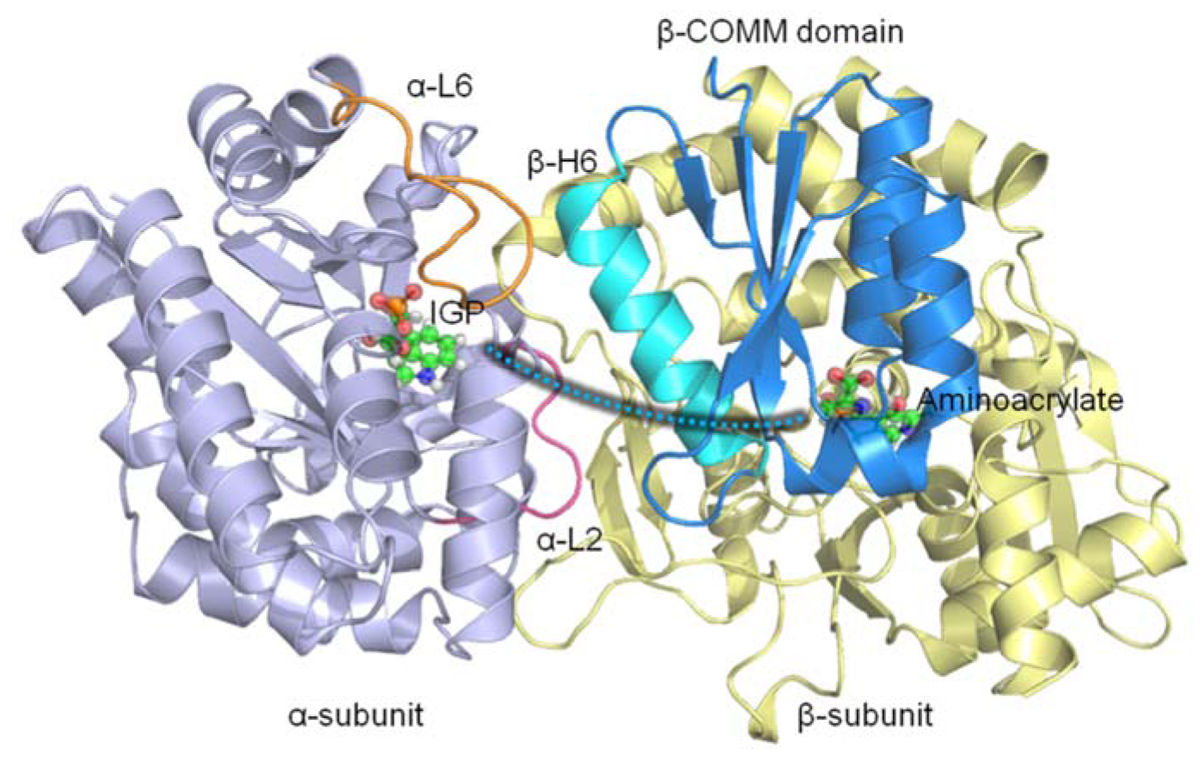
Overall structure and chemical reactions of tryptophan synthase (TRPS). TRPS is composed of an α- (purple) and β-subunit (yellow). The two ligands binding to each subunit are shown in bead representation. The open, partially closed, and fully closed conformations of the α-subunit are controlled by α-L2 (pink), α-L6 (orange), and β-Helix-6 of the COMM domain (cyan). The tunnel used to channel indole from the α-site to the β-site is marked as a cyan dashed shaded line.

**Figure 2:**
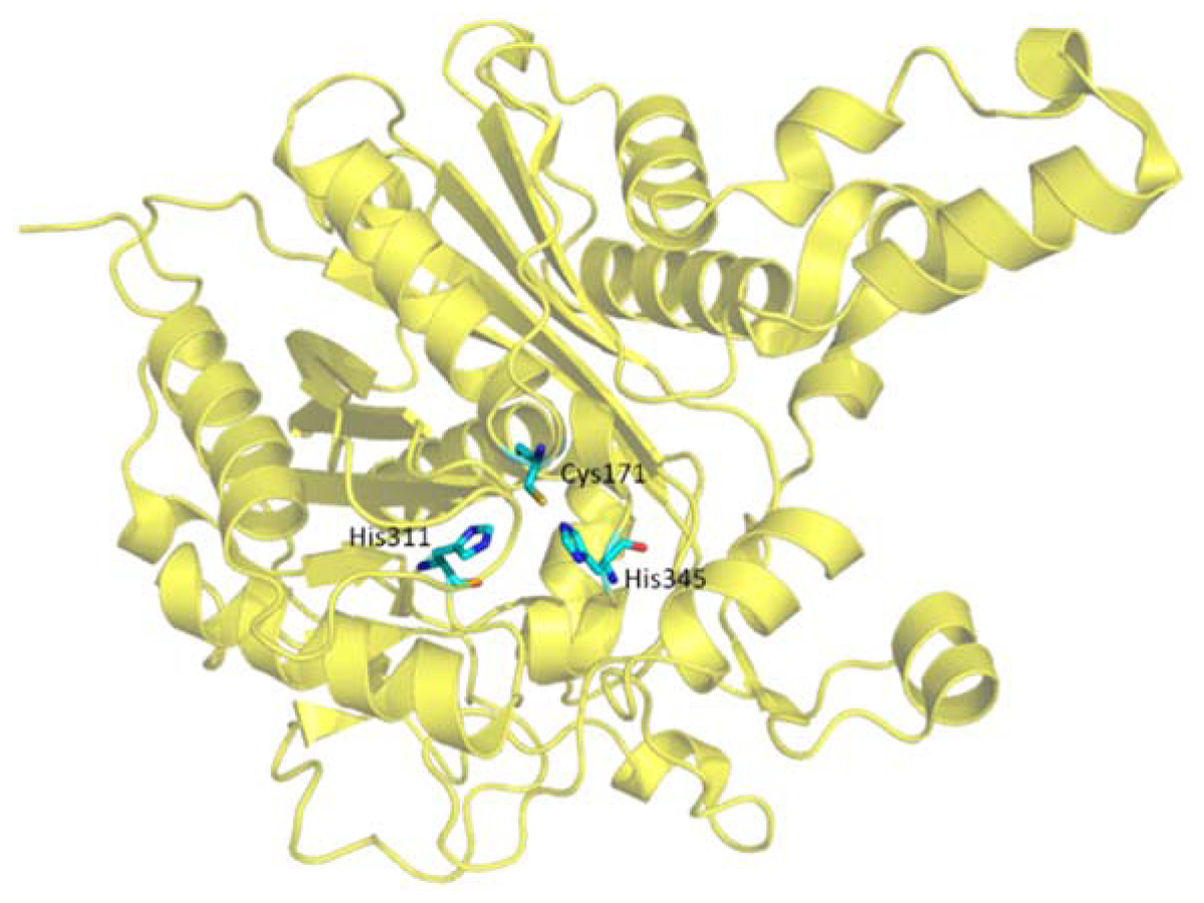
Overall structure of KasA. Three residues in the active site, Cys171, His311 and His345, important for catalysis are highlighted in bond representation.

**Figure 3:**
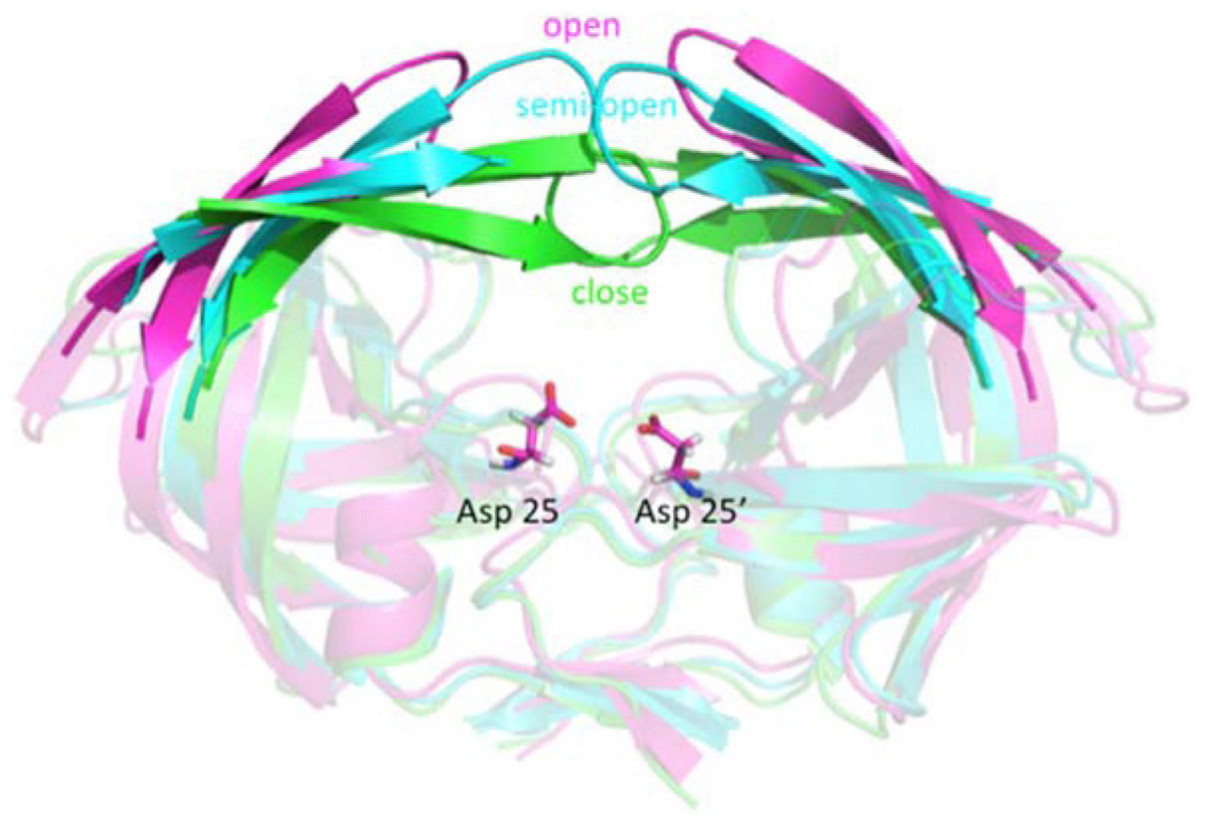
Structure of HIV-1 protease. Pink, cyan, and green indicate open, semi-open and closed flap conformation, respectively. The residues Asp-25 and Asp-25′, highlighted in bond representation, are associated with chemical catalysis and their protonation states have been investigated.

In this review, we discuss three well-known model systems, tryptophan synthase (TRPS) in Figure 1, β-Ketoacyl ACP synthase I (KasA) in Figure 2, and HIV-1 protease in Figure 3. The systems have been studied extensively for understanding large-scale protein motions, allosteric regulations and/or protonation states of key residues and ligands.

**Figure 4:**
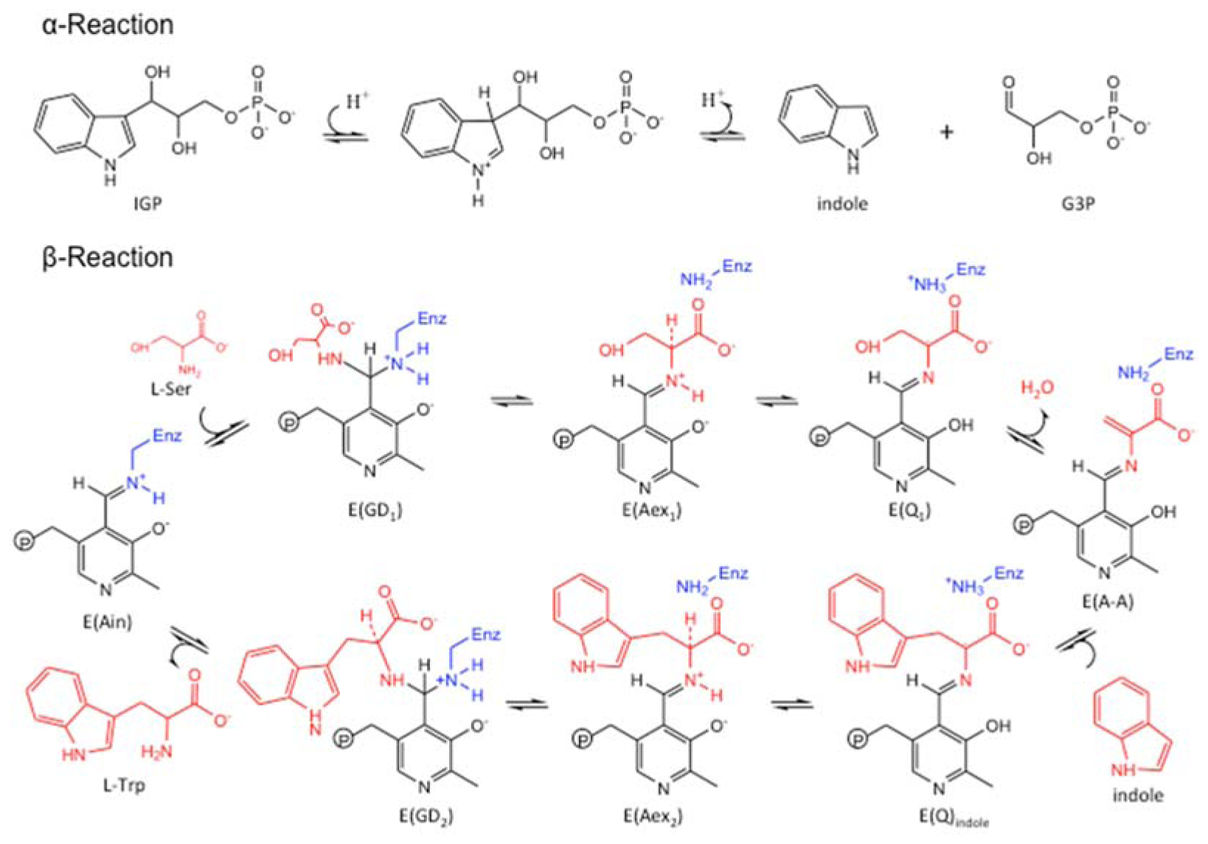
Overall chemical reactions in TRPS.

Bacterial TRPS is a bifunctional tetrameric αββα enzyme complex that catalyzes the last two reactions in the biosynthesis of L-tryptophan (L-Trp). In its α-subunit, the enzyme catalyzes the α-reaction, which involves cleavage of indole-3-glycerol phosphate (IGP) to indole and glyceraldehydes-3-phosphate (G3P) [10]. Indole then diffuses in the channel within the enzyme complex and is condensed with serine at the β active site (β-reaction) (Figure 4). This pathway involves at least nine different intermediates—E(Ain), E(GD_1_), E(Aex_1_), E(Q_1_), E(A-A), E(Q_2_), E(Q_3_), E(Aex_2_), and E(GD_2_)—formed from PLP and the reacting substrates (Figure 4) [11,12]. The αβ-reaction involves the following combination:

(1) α-reaction:→IGP→G3P+indole

(2) $$\beta \text{-reaction:}\to \text{Indole}+\text{L-Ser}\to \text{L-Trp}+H_{2}O$$

TRPS complexes with E(Q_3_) bound to the β-site; the complex readily reacts and is difficult to crystallize, so substrate analogues equivalent to the intermediates formed along the β-reaction pathway were widely studied. For example, the indole analogues indoline and 2-aminophenol (2AP) can rapidly react with E(A-A) to produce the long-lived indoline quinonoid E(Q)_indoline_, and 2AP quinonoid E(Q)_2AP_, respectively [13,14]. TRPS is a good model system for investigating PLP-dependent enzymatic mechanisms and for studying substrate channeling and allosteric networks that regulate protein functions.

**Figure 5:**
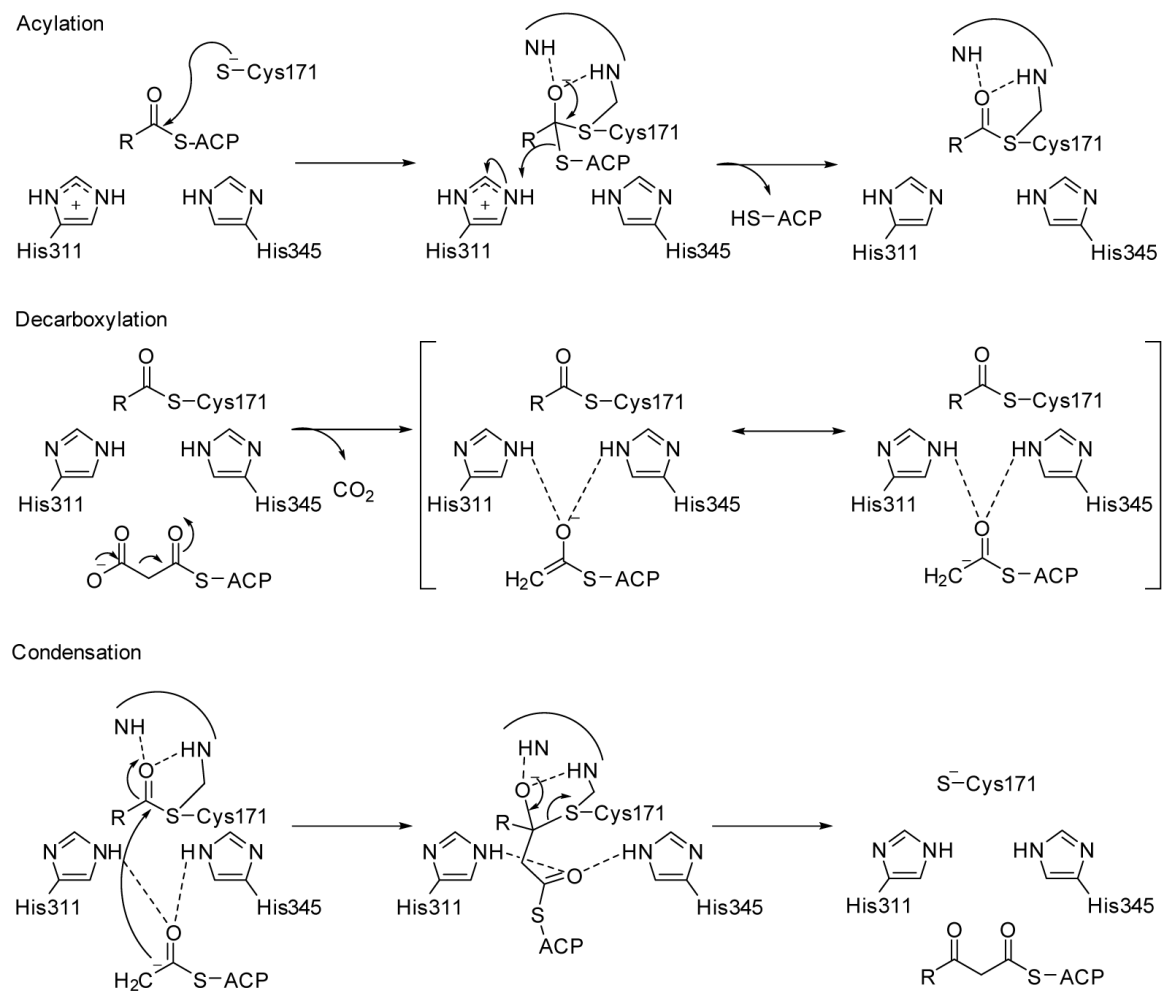
Catalytic mechanism of KasA.

β-Ketoacyl ACP synthase I (KasA) is a key enzyme in the survival of *mycobacterium tuberculosis* (*M. tuberculosis*), the causative pathogen of tuberculosis [15]. It has been proven that KasA is important in the biosynthesis of mycolic acid, which is one of the building blocks of the cell wall in *M. tuberculosis*. Therefore, KasA is a promising drug target in tuberculosis treatment. The catalytic cycle of enzyme mechanism consists of three steps, acylation, decarboxylation and condensation (Figure 5). First, Cys171 of the active site attacks and binds with acetyl chain delivered by acyl carrier protein (ACP). ACP is subsequently eliminated, and His311 is deprotonated in the acylation step. Decarboxylation of another ACP in the active site generates an enolate, which is stabilized by His311 and His345. The enolate tautomerizes to a carbanion that attacks the carbonyl carbon of acylated Cys171, which finally forms an elongated acyl chain. However, the enzyme mechanism has been debated and discussed [5], due to the multiple possible protonation states of its catalytic residues, Cys171, His311 and His 345.

HIV-1 protease is a dimeric aspartic protease that cleaves the premature polypeptide of HIV-1 and plays an essential role in viral replication [16–18]. Inhibitors targeting HIV-1 protease have been developed and represent among the major antiretroviral therapies for AIDS. Their development is considered a milestone success of structure-based drug design [19]. The motion in the flaps of HIV-1 protease plays a critical role in substrate binding and catalysis. Because of the intrinsic flexibility of HIV-1 protease, the free enzyme contains various flap conformations, usually termed semi-open, open, and closed [20,21]. Most apo structures show a semi-open flap conformation; however, the flaps must open for binding to a natural substrate. The protonation states of the two catalytically important aspartates, Asp-25 and Asp-25′, have been investigated to understand the catalytic mechanism and inhibitor recognition [22]. Because hydrogen atoms of Asp-25 and Asp-25′ can contribute to an important hydrogen bond, the protonation states of the residues need to be considered in structure-based drug design.

Recently developed molecular modeling tools have advanced our knowledge of the atomistic details of enzyme dynamics and catalysis. Through combining experimental techniques such as NMR, single molecule spectroscopy and computational tools, one can not only unveil the protonation states of catalytically significant intermediates of enzymes, which serves as a key component to reveal the catalytic mechanism, but also examine large-amplitude conformational movements of proteins in greater details. In this review, we discuss the applications of molecular dynamics (MD) simulations to investigate atomistic information of TRPS, MD and multi-scale quantum mechanics/molecular mechanics (QM/MM) methods to investigate protonation states of catalytic key residues of TRPS and KasA, and Brownian dynamics (BD) simulations to model large-scale conformational changes in HIV-1 protease for substrate recognition.

## 2. Computational Tools

### 2.1. Atomistic Molecular Dynamics Simulations

MD simulations, first developed over 30 years ago [23,24], have advanced from a method to simulate movements of several hundreds of atoms to a widely used way to study the structure and dynamics of macromolecules such as proteins or nucleic acids. Simulation of systems with ~50,000–100,000 atoms are now routine. The simulated system can be represented with different levels of detail, among which atomistic representations can best reproduce the actual systems; coarse-grained representations are useful with large systems or long simulations [25].

**Figure 6:**
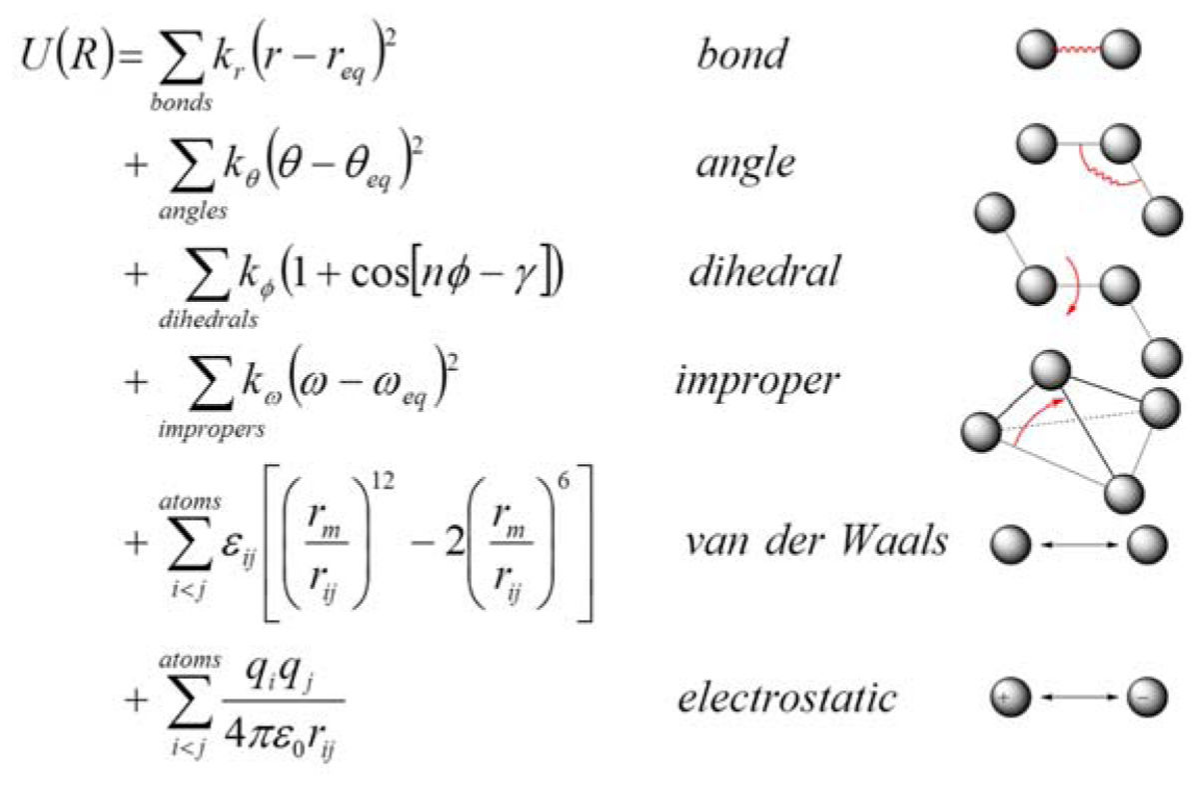
Classical force fields used for MD simulations: (**Right**) potential energy terms in a force field; and (**Left**) energy function used to derive atomic forces for molecular movement. *r* is the bond length; *θ* is the atomic angle; *φ* is the dihedral angle; *ω* is the improper dihedral angle; *r_ij_* is the distance in between atom *i* and *j; k_r_*, *k_θ_*, *k_φ_*, and *k_ω_* are force constants; *r_eq_*, *θ_eq_* and *ω_eq_* are equilibrium positions; the dihedral term is a periodic term characterized by a force constant (*k_ω_*), multiplicity (*n*), and phase shift (γ); *ε_ij_* is related to the Lennard–Jones well depth; *r_m_* is the distance at which the potential reaches its minimum; *q_i_* and *q_j_* are the charges on the respective atoms; and *ε_0_* is the dielectric constant.

In MD simulations, an initial model of the system is prepared from nuclear magnetic resonance (NMR), crystallographic, or built by homology-modeling when experimental data are not available. Different solvation models, including both explicit and implicit representations, can be used for the simulation [26–34]. Once the simulation system is built, forces acting on each atom can be obtained by deriving equations with parameters such as equilibrium bond length or angle, partial atomic charge, and van der Waals atomic radii (called “force-field” [35,36]) (Figure 6). Forces arising from interactions occur from both bonded and non-bonded atoms. Chemical bonds, atomic angles and improper angles are modeled by harmonic motions, and dihedral angles are modeled by using a sinusoidal function that approximates the energy differences between eclipsed and staggered conformations. Non-bonded forces include van der Waals interactions, modeled using the Lennard–Jones potential, and electrostatic interactions, modeled using Coulomb’s law.

Forces acting on individual atoms are then used for the calculation of accelerations and velocities with classical Newton’s law of motion. Therefore atom positions are updated after each time step. Here comes one principle challenge that limits the performance of atomistic MD simulation. To avoid possible collision, the simulation time is advanced, often by only 1 or 2 fs. The time step needs to be shorter than the timescale of bond stretching, the fastest motion in the molecule movements. Tens to hundreds of nanoseconds are currently standard simulation lengths for systems with ~100,000 atoms. However, the achievement of adequate sampling of conformational states may require at least microsecond-long simulations, which would cost much more computational time. While for coarse-grained simulation, which involves a more simplified representation of the system, we can use much larger time steps to greatly extend the length of simulations, in which case the long time length simulation is obtained at the cost of the accuracy including neglecting detailed atomic movements. The good news is, with the rapid development of computational technology and algorithms, we have greatly improved the performance of atomistic MD simulations.

During the past decade, molecular dynamics simulations performing on computer clusters or supercomputers using hundreds of processors in parallel has become very common. With the Message Passing Interface (MPI), by using multiple processors for one calculation task simultaneously, we can largely reduce computation time [41]. The most popular simulation software packages (AMBER [37], CHARMM [38], GROMACS [39], NAMD [40] or TINKER [43]) have long been compatible with MPI. In recent years, the use of graphical processing unit (GPU) cards to accelerate calculations is a major breakthrough in computational simulation field. With the use of GPU cards, which include many arithmetic units working in parallel, MD simulations can be accelerated by tens of times, so a single PC with such a card has the power similar to that of a cluster of workstations with multiple processors. Many major MD codes have already been rewritten to incorporate GPUs [42,44]. Moreover, with the Anton machine that specifically designed for MD simulations [45,46], it is possible to study protein dynamics on a millisecond timescale [47].

Another strategy to improve of efficiency of MD is through enhanced MD simulations. Various enhanced MD simulation methods have been developed over the past decade, among which coarse grain is one of the early approaches [48–50]. Other approaches like hyperdynamics [51], accelerated MD [52], RaMD-db [53] and Gaussian accelerated MD (GaMD) [54] accelerate the simulation by raising the potential energy well to lower the energy barrier. Another group of enhanced MD methods improve sampling by employing additional forces to the region of interest, including steered MD [55,56], target MD [57] and self-guided Langevin dynamics (SGLD) [58,59]. There are also other important MD based simulation methods that can enhance sampling. LowModeMD [60] uses low frequency modes from normal mode analysis to guide MD simulation. Transition interface sampling [61] performs intensive sampling at the transition interface, and obtains the kinetics information from probability. Replica exchange molecular dynamics (REMD) [62,63] makes configurations at high temperatures available to the simulations at low temperatures and vice versa, leading to a robust ensemble sampling both low and high energy configurations. Both computational technologies and enhanced MD simulations have made great progress enhancing computational calculations, it is anticipated that future development will continue improving the efficiency and lead to a new chapter for MD simulation.

### 2.2. Quantum Mechanics/Molecular Mechanics (QM/MM) Method

Though MD is useful in simulating both local atomistic movements and large-scale protein motions, it lacks the ability to model chemical reactions that involve changes in electronic structures, such as bond breaking, bond forming and charge transfer. Quantum Mechanics (QM) would be the perfect method to describe the changes of electronic structures; however, the large computational demand for electronic structure calculation limits the use of QM methods to only small systems with several hundred atoms. To study large biomolecules, Warshel and Levitt first came up with a hybrid quantum mechanics/molecular mechanics (QM/MM) approach in 1976 [24], which is further developed and evaluated by Karplus by coupling semi-empirical QM methods to MM force field [64]. These three together won the 2013 Nobel Prize in Chemistry for “the development of multiscale models for complex chemical systems”.

The QM/MM methods combine the accuracy of QM and speed of MM; thus allowing for the study of chemical reactions in enzymes. The general idea of QM/MM methods is to divide a system into an inner QM region that covers substrates and residues in the enzyme active site, and an outer MM region that includes the rest part of the protein and solvent. If water molecules also play critical roles in catalysis, explicit water molecules in the active site are also included in the inner region. The inner region is treated by a highly accurate QM method, and the outer region is treated by an inexpensive MM method. Therefore, the total potential energy for the system is a sum of MM energy terms, QM energy terms and QM/MM coupling terms:

$$V_{\text{QM}/\text{MM}}=V_{\text{QM}}(\text{QM})+V_{\text{MM}}(\text{MM)}+V_{\text{QM-MM}}(\text{QM}+\text{MM})$$

The QM/MM method is now widely used to model biomolecular systems [65–67], as well as other systems such as inorganic/organometallic [68,69], solid-state [70,71] and explicit solvent systems [72,73]. Moreover, combined with X-ray crystallography or NMR, QM/MM methods are also useful in the refinement of protein structures [74–77].

### 2.3. Combining Calculation, X-ray Crystallography and Solid-State NMR for Determining Protonation States

Using X-ray crystallography to determine high resolution protein structures is the first step to provide atomistic details for understanding enzyme function and mechanism. Even at high resolution, protonation states are difficult to be determined by solely X-ray crystallography. Therefore, we use the chemical shift in NMR spectroscopy as an extremely sensitive probe of the chemical environment to determine charge states and electrostatic fields at enzyme active sites. With NMR spectroscopy, isotropic chemical shifts can be used in two specific ways: (1) for directly reporting the chemical state of a probe atom to examine the protonation states or hybridization state at that site; or (2) analyzed for reporting the chemical and structural environment surrounding a probe nucleus. Notably, although solution NMR may not be applicable to proteins with more than several hundreds of residues, solid-state NMR can be used with large protein systems such as TRPS. Solid-state NMR spectra can be obtained on microcrystalline protein samples, prepared basically under the same conditions used to determine X-ray crystal structures [78]. Computational chemistry plays a key role in achieving a high level of structural details for determining protonation states. Typical X-ray and NMR experiments mostly show a protein structure in its static state, and molecular modeling tools are used to illustrate a dynamic view for protein motions and hydrogen bond networks. Although NMR chemical shifts are directly available from experiments, a proton may exchange between multiple ionizable sites. To more accurately predict the protonation states in the equilibrium, a proton can be placed in multiple sites, and *ab initio* calculations are then carried out for first-principle predictions of NMR chemical shifts. This calculation allows for further judging and ranking potential protonation site(s) based on their agreement with experiments [79].

### 2.4. Coarse-Grained Brownian Dynamics Simulations

Coarse-grained (CG) models for proteins have become popular in recent decades because of the need for modeling large-scale and/or long-time-scale protein motions such as protein folding or large-amplitude conformational fluctuations [80,81]. The CG model and BD simulations are also widely used approaches to investigate ligand–protein association processes. Although the association step is not discussed in this review, forming a substrate-enzyme complex is the very first step for any chemical reaction to happen in the active site of an enzyme. Typically a CG model uses one to six interacting centers (beads) to present each amino acid. A CG model such as a MARTINI CG force field can be a generic force field and the parameter set can be applied to most proteins [82]. Other CG parameters may be protein-dependent [83]. For example, Tozzini and co-authors developed a CG model for HIV-1 protease [84,85]. All the HIV-1 protease X-ray structures available in the Protein Data Bank were included in a statistical set, and the force-field parameters were derived by analyzing the set with the Boltzmann inversion procedure. The potential energy function is a sum of five kinds of interactions:

$$U=U_{\text{bond}}+U_{\text{angle}}+U_{\text{dihe}}+U_{\text{vdw}}+U_{\text{elec}}$$

**Figure 7:**
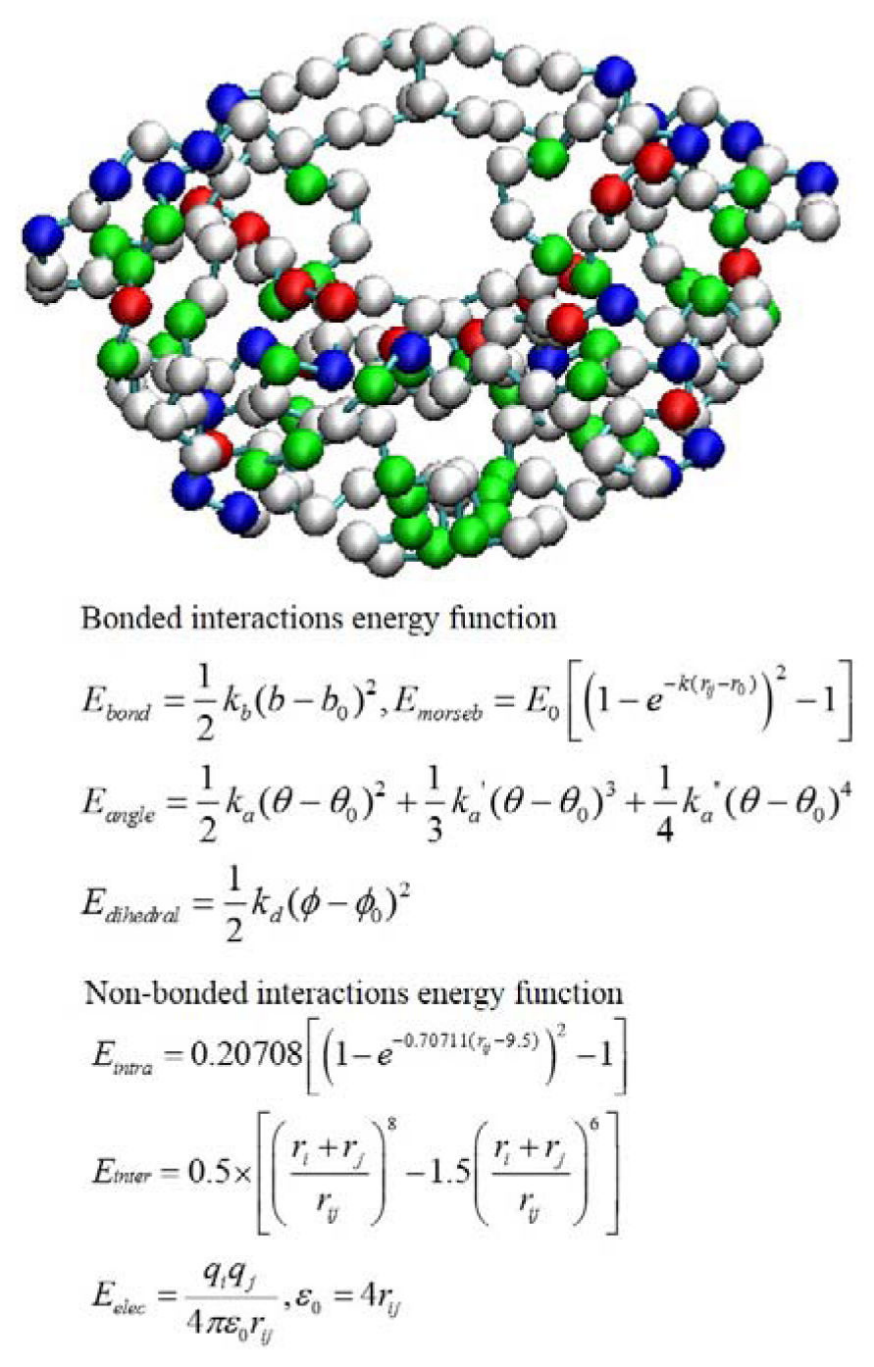
Representation of HIV-1 protease in the coarse-grained model and energy function of the force field. (**Top**) Each bead represents a residue, and positively charged, negatively charged and polar residues are in blue, red and green, respectively. (**Bottom**) The potential energy function of the force field for BD simulations, where *r* is the bond length; *θ* is the atomic angle; *φ* is the dihedral angle; *r_ij_* is the distance in between atom *i* and *j; k_b_*, *k_a_*, *k_d_*, and E*_0_* are force constants; and *b*_0_, *θ_0_* and *φ_0_* are equilibrium positions. *r_i_* and *r_j_* are the bead radius, and *q_i_* and *q_j_* are the charges on the respective bead. *ε*_0_ is the dielectric constant. E*_morseb_* is the Morse potential, a term for computing the bond energy between two consecutive beads. E*_intra_* and E*_inter_* are non-bonded interactions for *r_ij_* < 8 Å and > 8 Å, respective. Detailed parameters are in ref [85,88].

The representation of the one-bead model and the force-field functional forms are shown in Figure 7. The CG model has been implemented in the University of Houston Brownian Dynamics (UHBD) simulation package and the Reduced Molecular Dynamics (RedMD) simulation package [86,87].

Because microseconds or longer simulation lengths may be required to sample and obtain reasonable statistics for large-scale protein motions, BD simulations are used to further accelerate the simulations. Using the BD algorithm, we solved the Langevin equation of internal motion in the overdamped limit. This Ermak–McCammon equation is then used to gives a Brownian trajectory [89]. During the simulation, the time step dt may be set to 10–100 fs, which is significantly larger than with atomistic MD simulations, where time step is usually set to 1–2 fs. Hydrodynamic interactions are usually included in BD simulations, and this term is essential to simulate molecular systems under flow. However, calculating hydrodynamic interactions is time-consuming, and this term may be ignored if the hydrodynamic effects are negligible in modeling conformational fluctuations of a protein.

## 3. Examples of Modeling Enzymes and Substrates

### 3.1. TRPS: A Model System for Allosteric and Network Regulation in Enzyme Catalysis

**Figure 8:**
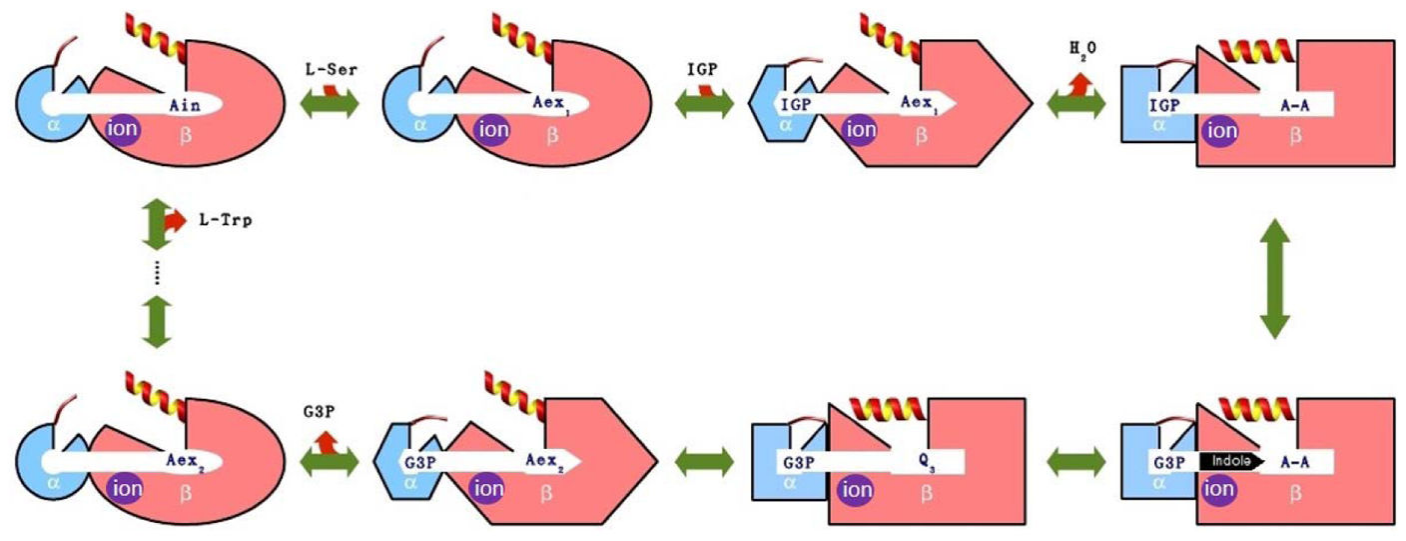
Cartoon representation of synergistic regulation in TRPS. The α- and β-subunit are represented by blue and pink, respectively. Different shapes represent different protein conformations, and α-L6 (thin line), and β-Helix-6 (helix) control open/closed of the active sites during different steps of chemical reactions.

Allosteric regulation is important in coordinating function and chemical reactions in many enzymes and protein complexes. A good model system that exhibits allosteric communication, synergistic regulation, and substrate channeling to enhance protein function is TRPS. As shown in Figure 8, TRPS shows synchronization of the α- and β-catalytic activities and conformational switching in its free and substrate bound states [90]. Atomistic MD simulations have been used to study the structure and dynamics of key residues for the α/β-dimer of TRPS and the motions of α-L6 and the COMM domain in TRPS with or without ligands. The MD simulations started from X-ray structures, the reference state. Because the flexible α-L6 loop is missing in all X-ray structures in the apo form, no open α-L6 loop structure is available experimentally. Therefore, the conformations of the open α-L6 were constructed by running MD simulations for a closed α-L6 loop conformation to open the loop [91,92]. With the absence of a ligand, the flexible α-L6 loop can sample open or partially closed conformations, but the loop tends to shift to fully closed conformations when its substrate is placed in the binding site. The fully closed conformations are induced by favorable ligand–protein interactions, which was not seen when the substrate is missing.

**Figure 9:**
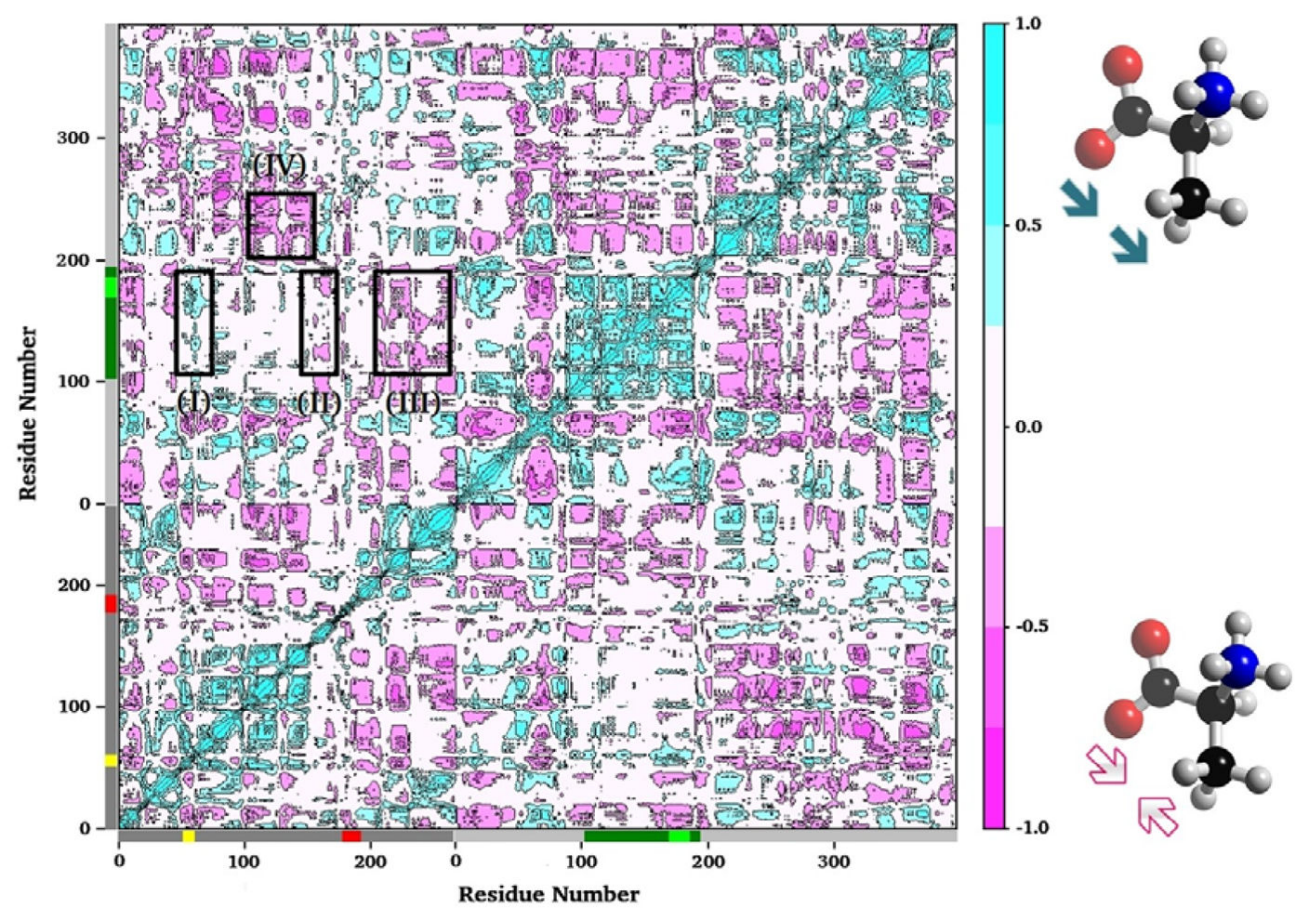
Plot of the residue–residue cross-correlation matrix. The plot was generated from a MD simulation of an apo TRPS by using the Bio3D program. Only Cα atom of each residue was used in the analysis. Negative values (pink) indicate anti-correlated motions that Cα atoms move along opposite directions. Positive values (cyan) represent correlated motions that Cα atoms move along the same direction. Square I shows the correlations between α-L2 and the COMM domain. Squares II and III show that residues of the two edges of α-L6 have anti-correlation motions with the COMM domain. Another example for correlated motions between the α/β interfaces is shown in square IV.

Post-analysis of MD simulations also reveals the most common pattern of protein motion and the dynamic correlations. For example, principal component analysis shows that α-L6 and α-L2 loops and the COMM domain have a strong tendency to move in concert (Figure 1). This synergistic movement is important because α-L6 and α-L2 facilitate the formation of the fully closed conformation to catalyze the α-reaction. In the meantime, the COMM domain receives the signal from allosteric communication to induce the fully closed conformation of the β-site and thereby enhance the overall rate of the α/β-reaction. The correlated motion that supports the experimental suggestions is shown in Figure 8, whereby a bound α-substrate induced a fully closed conformation of α-L6 and α-L2 loop and then further accelerated the COMM domain closure. A detailed cross-correlation matrix can be plotted with programs such as T-Analyst or Bio3D [93,94], which illustrates residue interactions during MD simulations. Correlations between pairs of residues are presented in cyan (correlated motion) or pink (anti-correlated motion) in Figure 9; correlated motions between α-L6 and α-L2 and the COMM domain are highlighted in squares. The COMM domain and α-L2 loop tend to move to the same direction (region I in Figure 9) and the motion of α-L6 is anti-correlated with that of the COMM domain (regions II and III).

**Figure 10:**
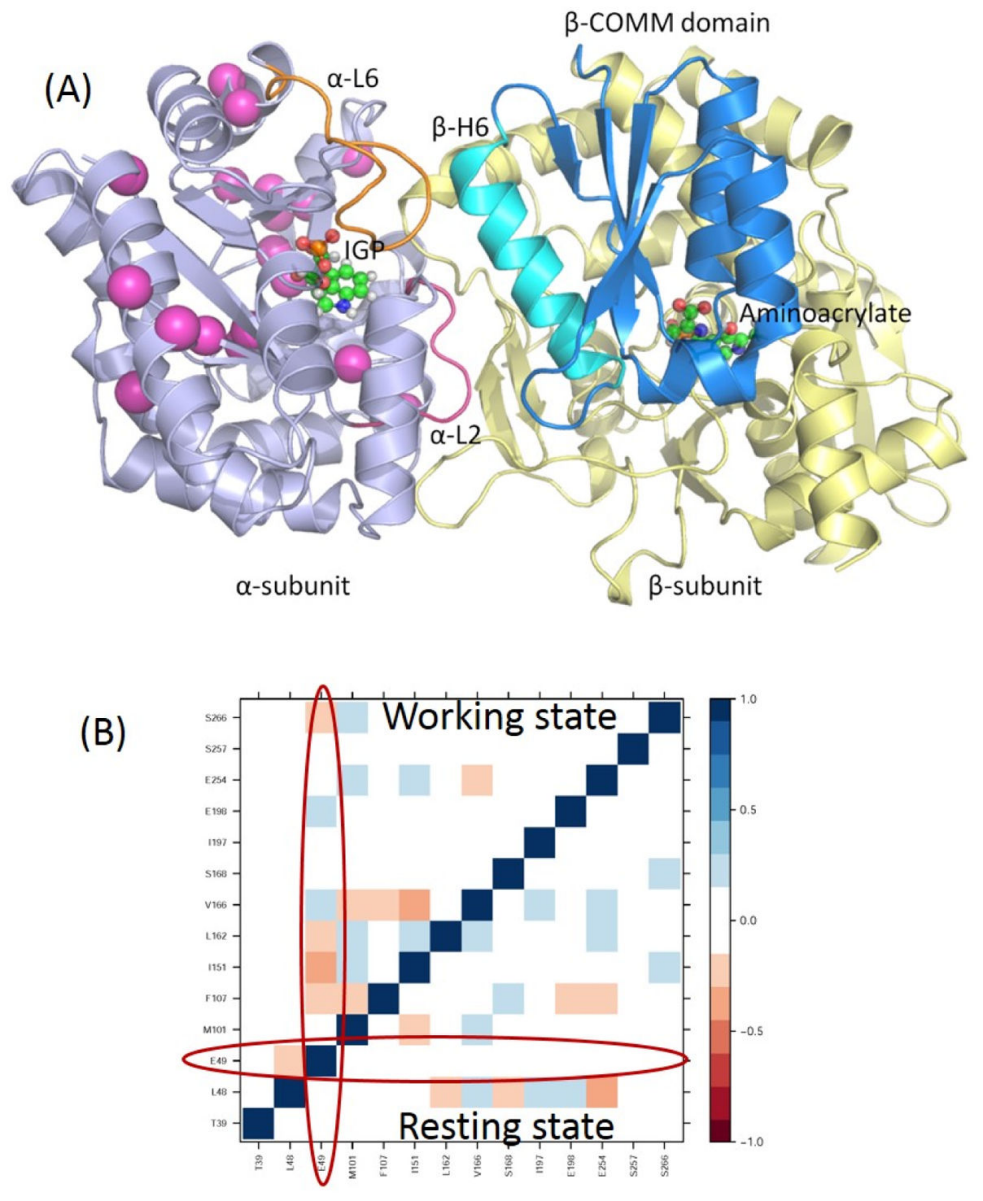
A cluster of residues of the TRPS α-subunit and their cross-correlation map: (**A**) residues identified by CHESCA that show correlated motions in the working state are presented as pink spheres; and (**B**) a cross-correlation map generated from a 100-ns MD simulation for the isolated α-subunit of TRPS from *Salmonella typhimurium.* The residues are shown in (**A**). The program T-Analyst was used to compute the side chain dihedral angles and their correlations with other residues. If a residue has more than one side chain dihedral angle, only the one closest to the protein backbone is used in the analysis. The lower triangle is for the ligand-free (resting) state, and the upper triangle is from the substrate-bound (working) state. The circles indicate the cross-correlations of Glu49 to other residues in the resting and working state, with no significant correlations with the residues in the resting state.

In addition to large amplitude loops and domain motions to open or close the active sites, some residues can form networks of correlated motions for chemical catalysis. NMR chemical shift covariance analyses (CHESCA) and MD simulations are powerful tools to detect such amino acid networks when substrates or products are absent (resting state) and present (working state) in the active site [95–97]. We investigated TRPS in its resting and working states to identify the correlation networks from a cluster of residues. We found that a cluster of residues can form a strongly correlated network in its working state, but the same correlation does not exist in its resting state (Figure 10). Mechanistic studies suggested that Glu49 of α-subunit is a key residue for chemical reaction. NMR studies also indicated that Glu49 and a cluster of other residues formed a network motion in the working state for the α-subunit of TRPS from *Escherichia coli*. Figure 10A shows a cluster of residues that form a correlation network: Glu49, Leu48, Thr39, Val197, Gly98, Met101, Phe107, Ile151, Ile166, Ala167, Ser168, Gly170, Ala198, Ala205, Aal254, Val257 and Thr266. Post-analysis for a 100-ns MD simulation for an isolated α-subunit of TRPS suggested that the correlated motions changes significantly between the working and resting state, which are consistent with the results from NMR experiments (Figure 10B). In particular, the catalytic important Glu49 shows considerable correlated motions with residues identified in the above cluster when the substrate is in the bound form (working state) but not in the resting state. Notably, X-ray structures show that Glu49 can stay at different locations, so proper positioning of Glu49 may play a role in regulating enzyme activity. This observation is consistent with results from NMR and MD that Glu49 performs highly correlated motions with other residues. These studies of TRPS demonstrated that examining the networks for catalysis and synergistic allosteric regulation is critical for fully understanding the functions of an enzyme or enzyme complex. The information may be useful for engineering enzymes and for other applications.

### 3.2. TRPS: How Protonation States Affect Protein Dynamics and Catalysis

**Figure 11:**
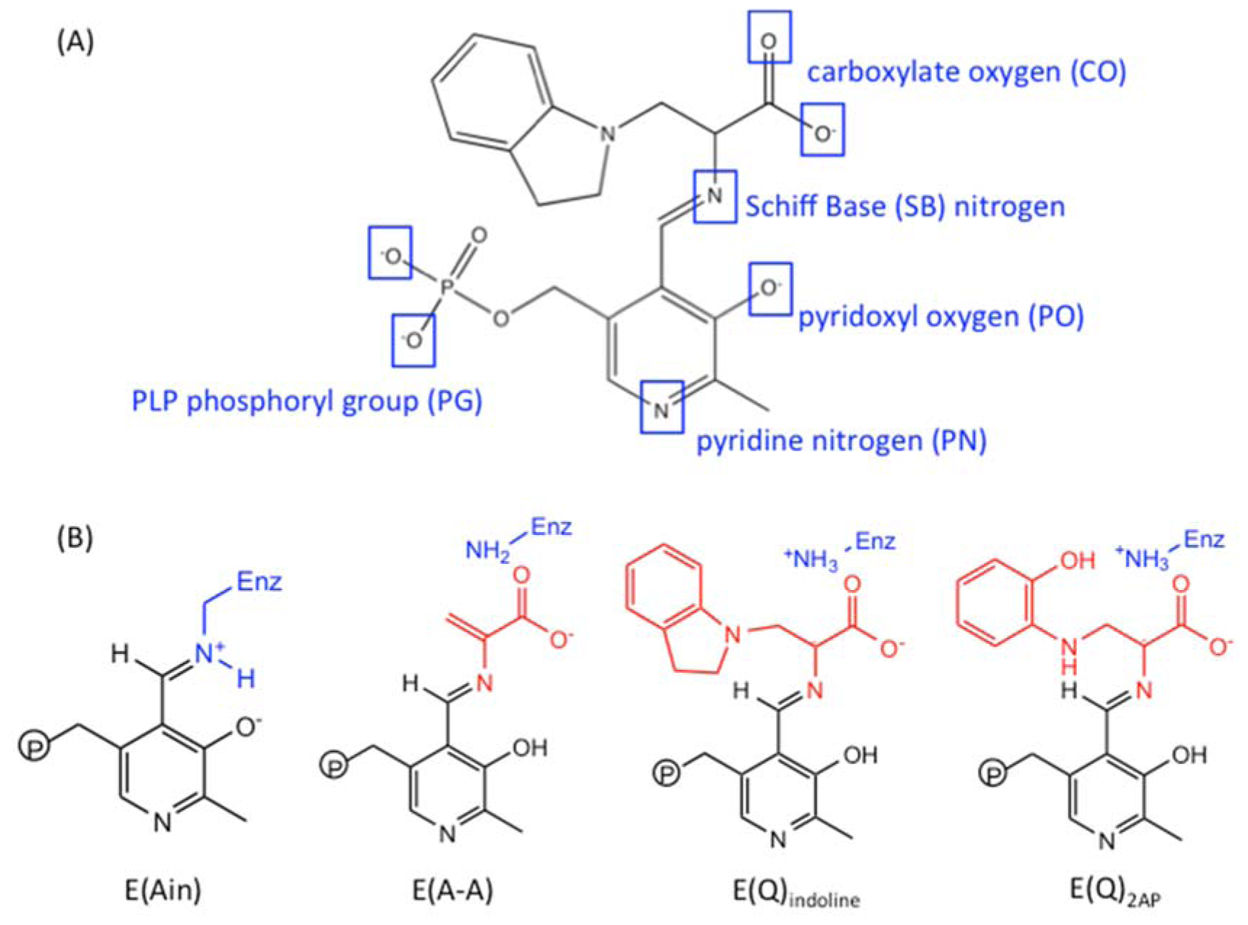
(**A**) Potential sites of protonation on an indoline quinonoid substrate; and (**B**) protonation states of four PLP substrates: E(Ain), E(A-A), E(Q)_indoline_ and E(Q)_2AP_.

Determining the protonation states of ionizable groups in β-reaction intermediates is critical for understanding the mechanisms of chemical reactions in TRPS catalysis since the relocation of a single proton is enough to promote or initialize a chemical reaction in the enzyme active site. On each PLP-bound intermediate, the protonation states play a crucial role in enhancing or weakening the attractions between the enzyme and substrate at six different locations: the phosphoryl group (PG), pyridoxyl nitrogen (PN), pyridoxyl oxygen (PO), Schiff Base (SB) nitrogen, and both carboxyl oxygens (COs) of the L-Ser substrate (Figure 11A). Combined studies of solid-state NMR and MD simulations have shown that the proton tends to stay at the SB nitrogen in the E(Ain) intermediate, whereas the proton transfers to PO in the E(A-A), E(Q)_indoline_, and E(Q)_2AP_ state. Moreover, the locations of PG, PN and both COs are all deprotonated during most of the lifetime [98,99]. The preference of protonation states in the four β-intermediates is shown in Figure 11B.

The catalysis of TRPS involves a series of proton transfers. Protonation or deprotonation of atoms at the catalytic site or the substrate directly affects structural stability, conformational changes and enzyme activity. For example, in the E(A-A) system, the proton at the PO helps stabilize the substrate in the binding site, so that the PG can form H-bonds with several nearby residues; the PN forms interactions with the β-hydroxyl group of the Ser377 side chain; and the CO atoms interact with the hydroxyl group of the Thr110 side chain and the backbone nitrogens of Gly111 and His115, which keeps the TRPS-E(A-A) complex in a closed conformation of the β-subunit, with no water molecules near the PN, PO, SB nitrogen, and COs. However, the simulations of the proton at the COs, SB nitrogen, PO and PG indicate that the weaker interactions between the COs and nearby residues result in the fluctuation of other residues around the substrate. As well, the H-bonds formed between the PO and Gln114 cause the opening of the β-site, so the solvent moves from bulk solution to the substrate binding site. Thus, changing the location with a single proton not only affects the stability of the local binding site but also alters the overall protein dynamics and molecular motions.

The proton at the PG and PN position has been widely investigated in PLP substrates. Until today, all evidence shows that in most systems of PLP catalysis, the PG is deprotonated. In addition, the PG binding sites are highly conserved—the oxygen atoms of the PG form stable H-bonds with a series of backbone Gly residues, which become major contacts between the PLP enzyme and substrate [100]. The protonated PG would result in a missing H-bond between the carboxylate group of L-Ser and Thr110/His115 and further affect the movement of the Gln114 loop. Thus, this would result in the opening of β-subunit from the original closed conformation. Similar effects have been identified with the change in protonation states at PN. Although the protonated PN has been extensively accepted because of a zwitterionic structure in coenzyme for stabilizing resonance carbanionic intermediates [101–103], recent studies suggest that some PLP enzymes, such as alanine racemase, O-acetylserine sulfhydrylase and TRPS, allow the PN in an unprotonated form [104–106]. Even without the proton at PN, a strong H-bond between the β-hydroxyl group of Ser377 and the PN can still form the carbanionic intermediates. In addition, charges at PN also vary along with the changes of the PN protonation state. The study of charge distribution at the PN shows that stronger negative charge, such as −0.9e, helps to stabilize the carbanionic intermediates as well as the protein conformations near the β-active site.

### 3.3. KaxA: Using QM/MM Methods to Determine Protonation States of Key Residues in Chemical Reactions

**Figure 12:**
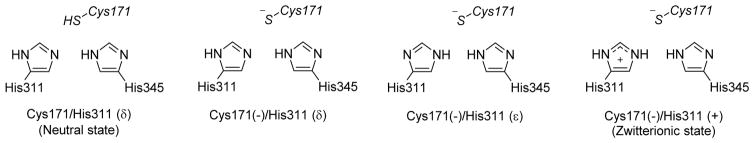
Four possible protonation states of catalytic residues of KasA.

The protonation states of catalytic residues, Cys171, His311 and His 345, of KasA are important in enzyme activity and understanding the catalytic mechanisms. Lee and Engels have been using QM/MM methods to investigate the reaction mechanisms [107–109]. The three catalytic residues can have total eighteen possible protonation states. From multiple X-ray crystal structures, it can be inferred that His345 is Nε protonated since Nδ forms a hydrogen bond with backbone amide of Ile347 as a hydrogen acceptor, which narrows down the number of possible protonation states to six. Among the six, Cys171/His311(ε) and Cys171/His311(+) can be excluded because they are unable to attack the substrate. Thus, researchers end up with four possible protonation states at the resting state of KasA (Figure 12). Since Lys340/Glu354 pair is at the active site and its protonation states, either neutral or zwitterionic, may influence the result of QM/MM calculation, their protonation states are also investigated.

**Figure 13:**
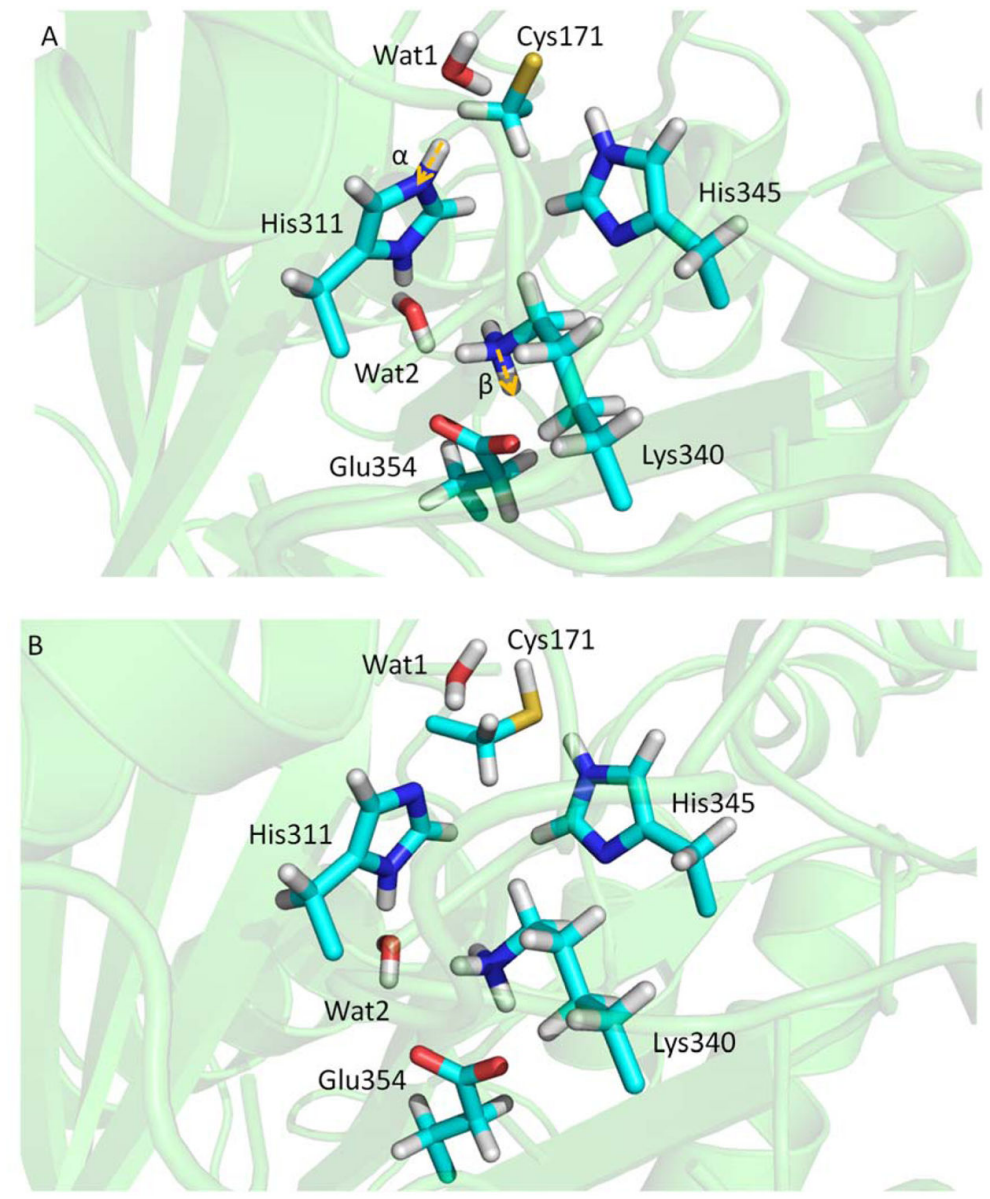
(**A**) Conformations of key residues of KasA in the resting state with protonation Cys171(−)/His311(+); and (**B**) conformations of key residues of KasA in the resting state with protonation Cys171/His311(δ).

QM/MM calculation was first conducted for the most probable protonation state, the zwitterionic state, Cys171(−)/His311(+). The QM part comprised Cys171, His311, Lys340, Glu354 and water molecules Wat1 and Wat2. The simulation was 100 ps with DFT (BLYP/6-31G**) for the QM region. The positions of protons along distances α and β can be measured (Figure 13). The distribution of distance α shows that neutral state Cys171/His311(δ) is preferred (~90%), while a population of ~10% indicates that Cys171(−)/His311(+) is less stable. Lys340/Glu354 pair (distance β) shows similar population for neutral and zwitterionic states. Free energy perturbation (FEP) was then conducted to compare the three protonation states, Cys171(−)/His311(δ), Cys171(−)/His311(ε) and Cys171(−)/His311(+), and pKa values were further derived from the computed free energy differences. Cys171(−)/His311(δ) can be ruled out due to its high acidity of the Nδ, while an equilibrium between Cys171(−)/His311(ε) and Cys171(−)/His311(+) can be predicted. Therefore, at the end of catalytic cycle, Cys171(−)/His311(ε) can change back to its initial neutral protonation state via zwitterionic state. By applying the QM/MM methods and FEP, a complete catalytic cycle has been obtained.

### 3.4. Investigating Large-Scale Conformational Changes in Enzymes: HIV-1 Protease

**Figure 14:**
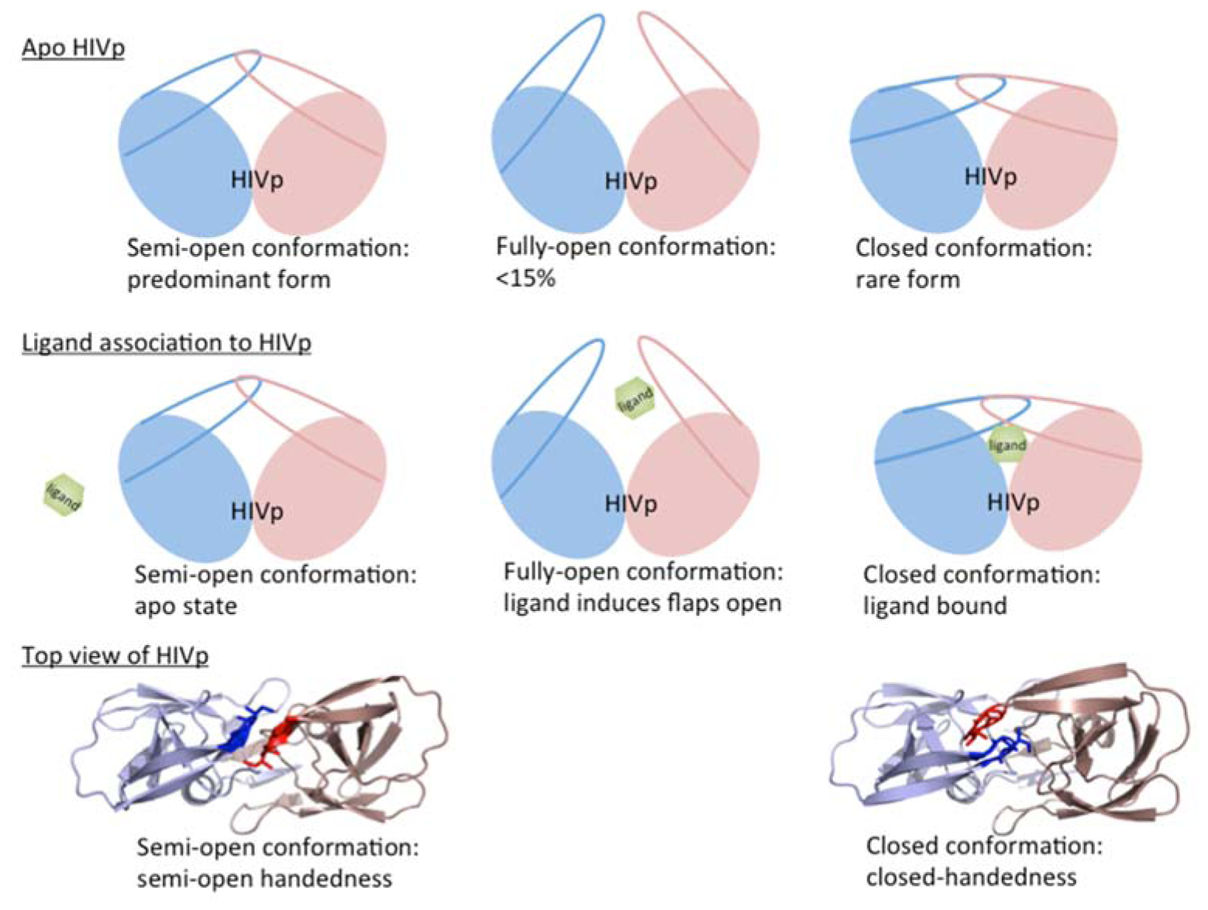
Cartoon representation of conformational rearrangements of HIV-1 protease. (**Top**) Three major HIV-1 protease conformations have been widely discussed. Semi-open conformations are the dominant form in the free state; however, fully-open and closed conformations are rare. (**middle**) A ligand binding to HIV-1 protease. The protein conformations change from semi-open, fully-open to closed forms. (**bottom**) The top view of HIV-1 protease shows that semi-open and closed conformations are with semi-open and closed handedness, respectively.

The dynamics of HIV-1 protease flaps are essential for substrate binding and catalysis. Atomistic MD and coarse-grained BD have been used to study the flap motions, flap rearrangements and substrate/inhibitor binding processes. HIV-1 protease is a C2-symmetric homodimer with the active site consisting of the residues Asp25/Asp25′, Thr26/Thr26′ and Gly27/Gly27′. The active site is gated by the two flaps that are extended beta hairpin loops and act as clamps to bind to a substrate or inhibitor. NMR studies suggested that the free HIV-1 protease has a substantial conformational change in the flap region, and the large-scale motion occurs on a micro- to millisecond (μs–ms) time scale [21]. Because HIV-1 protease is a well-studied system, hundreds of structures have been solved experimentally. Superimposing the experimental structures showed that most parts of the protein have similar conformations except for the flap regions. Three major conformations of HIV-1 protease have been widely discussed—fully-open, semi-open and closed conformation. Atomistic MD simulations suggested that the closed form is rare when a ligand is absent [110–112]. The semi-open conformations are the predominant form, and only <15% of apo HIV-1 protease appears with a fully open conformation (Figure 14 top). Both MD and BD simulations revealed that the flap elbows act as “hinges” to control the movement of the flexible regions, whereas the beta hairpin loops move relatively rigidly. Multiple 20-μs BD simulations with a coarse-grained model suggested that the average flap fully open and closed times are ~70 and 430 ns, respectively [85]. The orientation of flaps is called handedness. In addition to the large-scale conformational changes of the flaps, the handedness also varies along with the alternation of flaps motions from open to close. In general, semi-open and closed flap conformations are with semi-open and closed handedness, respectively (Figure 14 bottom).

In the apo protein, semi-open conformations, the predominant form of the flaps, still do not open widely enough for substrate association. Although the flaps can open spontaneously, the opening may be induced when a ligand is approaching to the enzyme, which results in an induced flap motion that may accelerate ligand binding [88,113,114]. After substrate/inhibitor binds to HIV-1 protease, the conformations of the flaps will switch from semi-open form with semi-open handedness to closed form with closed handedness (Figure 14 middle). Simulations complement existing observations from experiments and also provide more complete pictures of large-scale enzyme motions and residue rearrangements for ligand binding to help understand binding/catalysis mechanisms and for inhibitor design.

## 4. Outlook

Understanding enzyme dynamics and functions is important to building a complete picture of the catalytic mechanism and further help for enzyme design. Mechanistic studies of enzymes usually begin with investigating X-ray structures, which provide atomistic details for the first examination of chemical reactions and enzyme functions. With recent advances in the synergistic combination of NMR, MD simulations and *ab initio* QM calculations, we can now specify all atomistic information, including the protonation states, of an enzyme system. MD simulations and NMR further reveal dynamic features important to the enzyme functions. Although less directly observable, the allosteric regulation and correlated motions of a residue network are also involved in controlling substrate binding and chemical catalysis. Various computer modeling techniques, such as coarse-grained BD simulations discussed in this review, have been applied to complement atomistic MD simulations to sample large-amplitude and/or long-time-scale protein motions. The field of enzymology has evolved, and describing enzyme functions and catalysis is no longer based solely on simple organic chemical interactions. In addition, we need to consider protein motions involving communication between various residues and multiple regions of an enzyme complex. With more powerful computation tools and resources, molecular simulations will more significantly contribute to our understanding of enzyme systems and assist in enzyme engineering in the future.
